# The relationship between lactate/albumin ratio and prognosis in children with acute kidney injury

**DOI:** 10.1371/journal.pone.0329453

**Published:** 2025-08-01

**Authors:** Rui Zhang, Wenjin Feng, Kai Feng, Fengkai Sun, Xueyun Ren, Huabin Wang, Ru Yang

**Affiliations:** 1 Department of Pediatrics, Affiliated Hospital of Jining Medical University, Jining Medical University, Jining, China; 2 Shandong Provincial Key Medical and Health Discipline of Pediatric Internal Medicine, Affiliated Hospital of Jining Medical University, Jining, China; 3 Shandong University of Traditional Chinese Medicine, Jinan, China; 4 Department of internal medicine, Anju Sub-district Community Health Service Center, Jining, China; Osaka University of Pharmaceutical Sciences, JAPAN

## Abstract

Acute kidney injury (AKI) is a prevalent and critical complication in pediatric patients, severely influencing both survival outcomes and quality of life. This research seeks to evaluate the prognostic value of the lactate/albumin ratio (LAR) in predicting short-term outcomes for children diagnosed with AKI. A retrospective analysis was conducted using data from the Pediatric Intensive Care database, covering the period from 2010 to 2018. The relationship between the LAR and in-hospital mortality was explored using smoothing curve fitting, multivariate logistic regression, and Kaplan-Meier survival analysis. Receiver operating characteristic (ROC) analysis was performed to evaluate the predictive capability of LAR for in-hospital mortality. A total of 631 pediatric patients with AKI were included in this study. Smoothing curve fitting revealed a nearly linear positive association between the LAR and in-hospital mortality. Multivariate logistic regression analysis identified LAR as an independent predictor of in-hospital mortality (OR 2.58, 95% CI 1.85–3.59). Kaplan-Meier survival analysis showed that patients with a higher LAR had a significantly greater 30-day mortality rate compared to those with lower LAR values. ROC curve analysis demonstrated an area under the curve of 0.85 (95% CI 0.80–0.90). At the optimal cutoff value of 0.80, the sensitivity was 80.42%, specificity was 77.97%, and accuracy was 80.19%. These results suggest that LAR is a promising prognostic indicator of mortality in pediatric patients with AKI, and could serve as an early indicator of risk stratification.

## Introduction

Acute kidney injury (AKI) poses a significant clinical challenge in pediatric intensive care, marked by a rapid decline in kidney function, which leads to fluid imbalances, electrolyte disruptions, and serious metabolic disturbances. The incidence of AKI is steadily increasing worldwide, particularly among critically ill children in pediatric intensive care units (PICUs), with reported rates ranging from 20% to 40%, depending on the specific patient population and diagnostic criteria used [1,2]. Furthermore, the mortality rate among AKI patients remains high, especially in critically ill children, where prognosis is often unfavorable. This not only extends hospital stays and escalates medical costs but also substantially diminishes the quality of life and survival rates for affected children. If not diagnosed and treated promptly, AKI can rapidly advance to irreversible kidney failure, ultimately leading to death [3]. Given the limitations of conventional markers like serum creatinine and urine output in providing early and comprehensive prognostic insights, identifying reliable and accessible prognostic biomarkers remains crucial. Such prognostic markers could enhance early risk stratification and help tailor management strategies by potentially reflecting broader physiological disturbances in pediatric AKI patients.

The pathophysiology of AKI is characterized by a complex interplay of tissue hypoxia, inflammation, and metabolic dysfunction [4]. Lactate, a byproduct of anaerobic metabolism, serves as a well-established marker of tissue hypoperfusion and cellular stress. In the setting of AKI, renal hypoxia and microcirculatory impairment can cause elevated lactate levels, reflecting both local and systemic metabolic disruptions [5,6]. Additionally, serum albumin, predominantly produced by the liver, is crucial for maintaining colloid osmotic pressure, transporting various molecules, and providing antioxidant protection. In critically ill patients, especially those with AKI, albumin levels often decline due to multiple factors including malnutrition, liver dysfunction, systemic inflammation, and hemodilution secondary to fluid overload [7,8].

The lactate/albumin ratio (LAR) has emerged as a composite biomarker, combining these two physiologically important parameters. Compared to using either marker independently, LAR provides a more comprehensive evaluation of a patient’s metabolic state and level of physiological stress [9]. Recent research in adult populations has shown that an elevated LAR correlates with increased mortality risk in critical conditions such as sepsis and acute respiratory distress syndrome [10,11]. However, despite promising early data, the prognostic significance of LAR in pediatric AKI has yet to be fully investigated.

Currently, the prognosis of pediatric AKI is primarily assessed using conventional prognostic biomarkers like serum creatinine and urine output, although these indicators have notable limitations, such as delayed reflection of AKI prognosis and sensitivity to non-renal factors [12,13]. As a result, there is a critical need for novel, easily accessible biomarkers that can provide accurate prognostic information for children with AKI. LAR presents several advantages as a potential prognostic tool, including its routine availability in clinical practice, rapid result generation, and ability to simultaneously capture information on both metabolic and nutritional status [14].

This study seeks to investigate the association between the LAR and clinical outcomes in pediatric AKI, with a specific focus on hospital length of stay and final patient outcomes. We hypothesize that LAR may function as a useful prognostic biomarker, enabling the early identification of high-risk pediatric AKI patients who could benefit from more focused monitoring and intervention strategies.

## Methods

### Case source

The Pediatric Intensive Care (PIC) database, created by the Children’s Hospital at Zhejiang University School of Medicine, serves as a publicly available resource dedicated to pediatric critical care patients. It includes inpatient data for 12,881 pediatric patients who were admitted between January 1, 2010 and December 31, 2018. Data for this research were accessed from the PIC database starting on 04/01/2025. The project received ethical approval from the institutional review board at the Affiliated Hospital of Jining Medical University (2025C002). Given that this is a retrospective study with no impact on clinical decision-making, the requirement for patient informed consent was waived. Since all the information in the database has been de-identified, we were unable to access information that could identify individual participants during or after data collection.

### Inclusion and exclusion criteria

Inclusion Criteria: (1) Patients aged between 1 month and 18 years. (2) For patients with multiple admissions, only the data from their first hospitalization were considered. (3) We used the pROCK criterion, which defines AKI as an increase in creatinine levels of ≥ 20 µmol/L and ≥ 30% within 7 days [15]. Exclusion Criteria: (1) Patients in the neonatal intensive care unit. (2) Absence of chartevents data. (3) Missing lactate or albumin measurements.

### Data extraction, exposure, and outcome definitions

Demographic data, including age and sex, were collected. Laboratory results, the first recorded values obtained within 24 hours of admission to the PICU, included measurements of peripheral white blood cell count, platelet count, pH, bicarbonate, anion gap, serum sodium, potassium, glucose, activated partial thromboplastin time, lactate, and albumin levels. Comorbidities were identified based on diagnoses made within 48 hours of PICU admission, including anemia, liver dysfunction, hypertension, pneumonia, systemic inflammatory response syndrome (SIRS), and diabetic ketoacidosis. The definitions of these comorbidities are provided in S1 Table. In this study, missing data for all variables accounted for less than 10%. For imputation, missing values were replaced with the mean or median, depending on the nature of the variable.

The primary exposure factor was the LAR, defined as the ratio of lactate (mmol/L) to albumin (mg/L) measured within 24 hours of PICU admission. LAR was analyzed as both a continuous and categorical variable. Patients were categorized into low-LAR and high-LAR groups based on the median value of LAR. The primary outcome of the study was hospital mortality, defined as death from any cause during the hospitalization period. Secondary outcomes included 30-day mortality, length of stay (LOS) in the PICU, and total hospital LOS.

### Statistical analysis

Normally distributed continuous data were expressed as mean ± standard deviation, while data with non-normal distribution were presented as median (interquartile range). Differences between two groups were assessed using either the Student’s t-test or the Mann-Whitney U test, based on the distribution type. Categorical data were reported as frequency (percentage), with group differences compared using the chi-square test.

To explore the relationship between the LAR and hospital mortality, a generalized additive model was employed to fit a smoothing curve. Univariate and multivariate logistic regression analyses were conducted to further investigate this relationship. Covariates were adjusted using a stepwise modeling approach: Model 1 = LAR; Model 2 = Model 1 + demographic data (age and sex); Model 3 = Model 2 + laboratory test results; Model 4 = Model 3 + comorbidities. Laboratory test results were classified as binary variables (normal vs. abnormal) based on established reference ranges.

Kaplan-Meier survival curves for 30-day mortality were generated for different LAR levels, with comparisons made using the log-rank test. Receiver Operating Characteristic (ROC) curves were plotted to evaluate the predictive accuracy of LAR for hospital mortality in pediatric AKI patients. The Youden index (sensitivity + specificity – 1) was calculated, and the optimal cutoff value was determined by identifying the maximum Youden index.

To assess the robustness and internal generalizability of our primary findings regarding LAR’s predictive performance and optimal cutoff, we conducted two complementary sensitivity analyses. Firstly, we performed a Bootstrapping resampling procedure with 500 iterations on the original dataset. Secondly, to further examine the consistency of LAR’s performance in an independent subset, we randomly selected a 30% subsample (n = 190) from the total dataset. Within this subsample, we applied the optimal LAR cutoff, which was derived from the full dataset analysis, and then re-evaluated its predictive performance. Furthermore, we conducted a sensitivity analysis by excluding patients who received albumin substitution within the first 24 hours in the PICU or those with liver dysfunction, followed by repeating the logistic regression and ROC analyses. A *p*-value of <0.05 was considered statistically significant. All statistical analyses were performed using R software (version 3.5.3), MedCalc (version 18.2.1), and EmpowerStats (http://www.empowerstats.com).

## Results

### General characteristics of the enrolled patients

**Fig 1:**
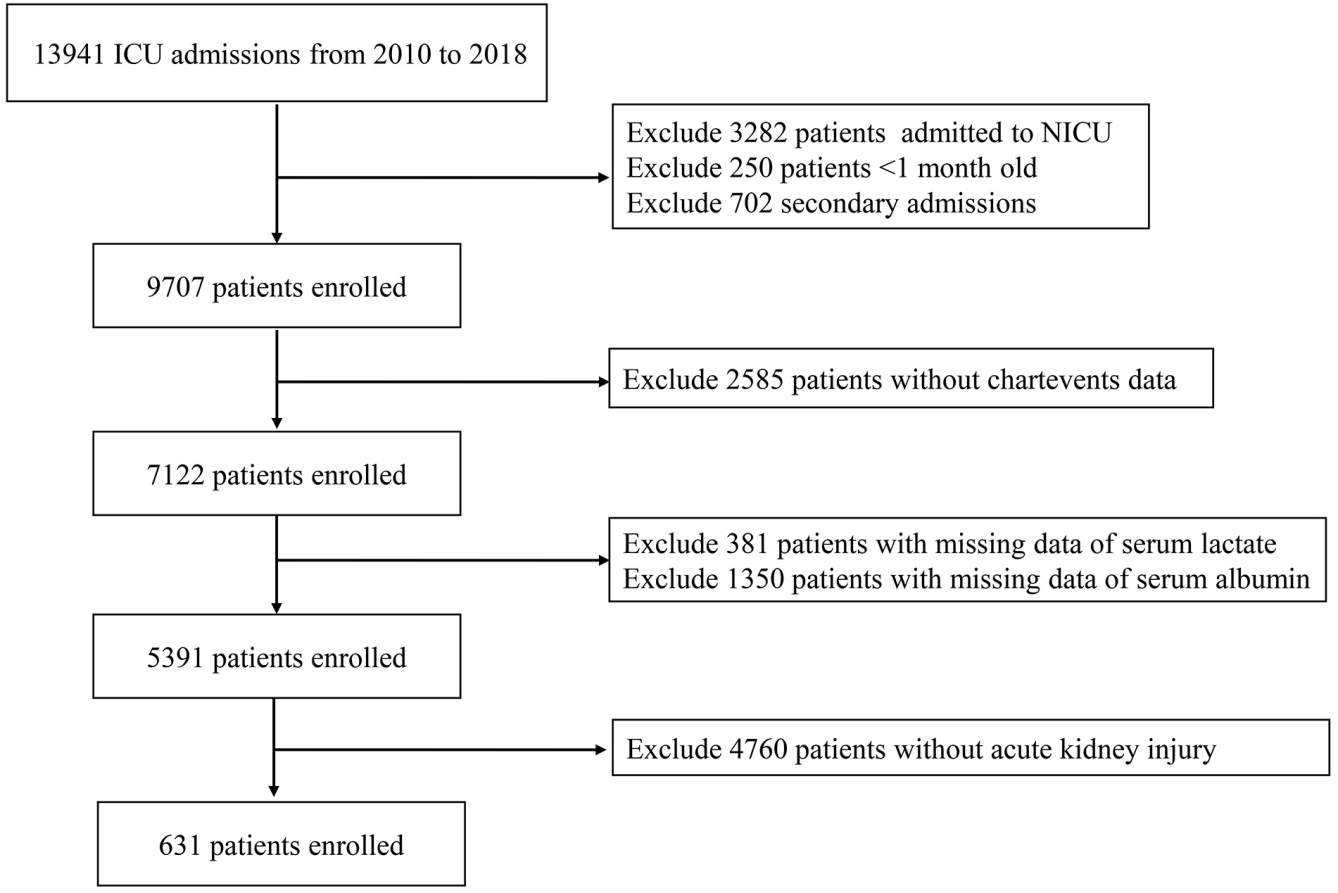
Flow chart for participants. ICU, intensive care unit.

The flowchart depicting the selection of study participants is shown in Fig 1. The baseline characteristics of the pediatric AKI patients are summarized in Table 1. A total of 631 pediatric patients with AKI were included in the study. Clinical data from the low-LAR and high-LAR groups were compared. As detailed in Table 1, the low-LAR group had a lower white blood cell count (9.7 vs. 11.8, *P* < 0.001), smaller anion gap (9.4 vs. 11.9, *P* < 0.001), and a lower proportion of liver dysfunction (8.9% vs. 26.9%, *P* < 0.001) when compared to the high-LAR group. In contrast, the low-LAR group had higher pH (7.4 vs. 7.3, *P* < 0.001), bicarbonate (21.9 vs. 20.3, *P* < 0.001), and glucose levels (7.9 vs. 10.2, *P* < 0.001) compared to the high-LAR group.

Regarding clinical outcomes, there was no significant difference in hospital LOS between the two groups (11.5 vs. 12.8, *P* = 0.061). However, the length of stay in the PICU was significantly shorter in the low-LAR group compared to the high-LAR group (3.0 vs. 4.5, *P* = 0.001). The 30-day mortality rate in the high-LAR group was 12.7 times higher than in the low-LAR group (16.5% vs. 1.3%, *P* < 0.001), and the hospital mortality rate in the high-LAR group was 8.8 times greater than in the low-LAR group (16.8% vs. 1.9%, *P* < 0.001).

**Table 1:** Baseline characteristics according to the tertiles of the lactate/albumin ratio (n = 631).

Variable	Lactate/albumin ratio		*P*-value
	Low (< 0.50) n = 315	High (≥ 0.50) n = 316	
**Age, months**	21.3 (8.6-56.9)	17.6 (5.0-64.0)	0.704
**Male, n (%)**	172 (54.6%)	167 (52.8%)	0.658
**Laboratory data**			
White blood cell, 10^9^/L	9.7 ± 5.0	11.8 ± 8.1	<0.001
Platelet, 10^9^/L	245.8 ± 128.1	234.7 ± 152.7	0.322
PH	7.4 ± 0.1	7.3 ± 0.1	<0.001
Bicarbonate, mmol/L	21.9 ± 3.6	20.3 ± 4.5	<0.001
Anion gap, mmol/L	9.4 ± 5.2	11.9 ± 6.7	<0.001
Sodium, mmol/L	137.3 ± 4.9	137.7 ± 5.7	0.539
Potassium, mmol/L	3.7 ± 0.7	3.8 ± 0.9	0.218
Glucose, mmol/L	7.9 ± 3.2	10.2 ± 5.3	<0.001
APTT, s	41.1 ± 21.6	40.5 ± 21.3	0.741
**Comorbidities, n (%)**			
Anemia	217 (68.9%)	210 (66.5%)	0.514
Hypertension	45 (14.3%)	34 (10.8%)	0.181
Liver dysfunction	28 (8.9%)	85 (26.9%)	<0.001
Diabetic ketoacidosis	24 (7.6%)	37 (11.7%)	0.082
Pneumonia	16 (5.1%)	26 (8.2%)	0.113
SIRS	147 (46.7%)	161 (50.9%)	0.282
**Clinical outcome**			
ICU LOS, days	3.0 (0.9-6.9)	4.5 (2.0-8.0)	0.001
Hospital LOS, days	11.5 (7.4-17.0)	12.8 (6.1-20.0)	0.061
30-day mortality, n (%)	4 (1.3%)	52 (16.5%)	<0.001
Hospital mortality, n (%)	6 (1.9%)	53 (16.8%)	<0.001

Data are expressed as the mean ± SD, median (interquartile range), or percentage; APTT, activated partial thromboplastin time; ICU, intensive care unit; LOS, length of stay; SIRS, systemic inflammatory response syndrome.

### Relationship between LAR and hospital mortality in pediatric AKI patients

**Fig 2:**
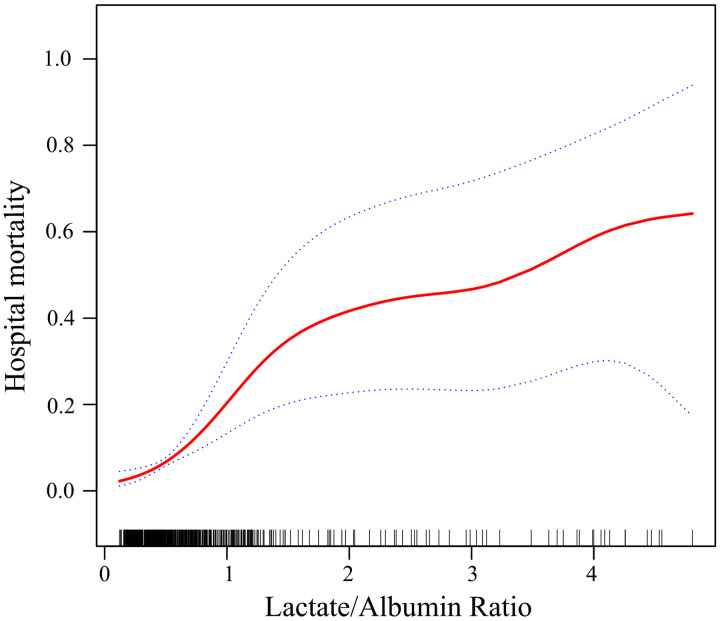
Using a smooth curve fitting analysis to examine the relationship between the lactate/albumin ratio and in-hospital mortality. Solid red line represents the smooth curve fit and blue bands represent the 95% CI. Adjusted for age, sex, laboratory tests, and comorbidities.

A smoothing curve fitting analysis demonstrated a positive correlation between LAR and hospital mortality in pediatric AKI patients (Fig 2). In the univariate logistic regression model, several factors were significantly associated with hospital mortality, including abnormal white blood cell count (odds ratios (OR) 2.00, 95% CI 1.17–3.43), abnormal pH (OR 4.17, 95% CI 2.41–7.22), abnormal bicarbonate (OR 3.21, 95% CI 1.70–6.07), abnormal sodium (OR 2.19, 95% CI 1.26–3.79), liver dysfunction (OR 4.77, 95% CI 2.72–8.36), diabetic ketoacidosis (OR 2.39, 95% CI 1.17–4.90), SIRS (OR 1.87, 95% CI 1.07–3.25), LAR (OR 2.89, 95% CI 2.23–3.75), and high LAR (OR 10.38, 95% CI 4.39–24.53) (Table 2).

**Table 2:** The unadjusted association between baseline variables and In-hospital mortality.

Variable	Statistics	OR	95% CI	*P*-value
Lactate/albumin ratio	0.50 (0.34-0.80)	2.89	2.23-3.75	<0.001
Lactate/albumin ratio (>0.50)	316 (50.08)	10.38	4.39-24.53	<0.001
Age				
<12 months	238 (37.72)	1.0		
12-60 months	233 (36.93)	0.44	0.23-0.86	0.016
>60 months	160 (25.36)	0.72	0.37-1.38	0.320
Gender (female)	292 (46.28)	0.98	0.57-1.67	0.934
WBC (< 4 or > 12, 10^9^/L)	245 (38.83)	2.00	1.17-3.43	0.012
Platelet (< 100, 10^9^/L)	80 (12.68)	1.89	0.96-3.75	0.067
PH (< 7.35 or > 7.45)	151 (23.93)	4.17	2.41-7.22	<0.001
Bicarbonate (<22 or >27, mmol/L)	346 (54.83)	3.21	1.70-6.07	<0.001
Anion gap (<8 or >16, mmol/L)	300 (47.54)	1.69	0.98-2.91	0.059
Sodium (< 135 or > 145, mmol/L)	169 (26.78)	2.19	1.26-3.79	0.005
Potassium (< 3.5 or > 5.5, mmol/L)	232 (36.77)	1.30	0.75-2.23	0.349
Glucose (< 3.6 or > 6.1, mmol/L)	477 (75.59)	0.78	0.43-1.41	0.409
APTT (>45, s)	177 (28.05)	1.25	0.70-2.21	0.457
Anemia	427 (67.67)	1.20	0.67-2.17	0.545
Hypertension	79 (12.52)	0.62	0.24-1.61	0.328
Liver dysfunction	113 (17.91)	4.77	2.72-8.36	<0.001
Diabetic ketoacidosis	61 (9.67)	2.39	1.17-4.90	0.017
Pneumonia	42 (6.66)	1.34	0.51-3.55	0.557
SIRS	308 (48.8)	1.87	1.07-3.25	0.027

APTT, activated partial thromboplastin time; OR, odds ratio; SIRS, systemic inflammatory response syndrome; WBC, white blood cell.

Data in the statistics column are presented as mean ± standard deviation or median (interquartile range) for continuous variables (lactate/albumin ratio), and as n (%) for categorical variables.

In the progressively expanded multivariate logistic regression models, high LAR remained significantly associated with increased hospital mortality and 30-day mortality (Table 3). When LAR was treated as a binary variable, the OR were as follows: Model 1 (OR 10.38, 95% CI 4.39–24.53), Model 2 (OR 9.85, 95% CI 4.16–23.34), Model 3 (OR 8.70, 95% CI 3.54–21.37), and Model 4 (OR 7.72, 95% CI 3.10–19.19). When treated as a continuous variable, the results were: Model 1 (OR 2.89, 95% CI 2.23–3.75), Model 2 (OR 2.95, 95% CI 2.25–3.86), Model 3 (OR 2.58, 95% CI 1.88–3.54), and Model 4 (OR 2.56, 95% CI 1.86–3.53). A similar pattern was observed for the association between LAR and 30-day mortality.

**Table 3:** Stepwise expansion of the multivariate logistic regression model (lactate/albumin ratio as a dichotomous variable or continuous variable).

	Lactate/albumin ratio (>0.50)		
	Odds ratio	95% confidence interval	*P*
**In-hospital mortality**			
Model 1	10.38	4.39-24.53	<0.001
Model 2	9.85	4.16-23.34	<0.001
Model 3	8.70	3.54-21.37	<0.001
Model 4	7.72	3.10−19.19	<0.001
**30-day mortality**			
Model 1	15.31	5.47-42.90	<0.001
Model 2	14.67	5.22-41.18	<0.001
Model 3	12.60	4.36-36.45	<0.001
Model 4	11.07	3.78-32.48	<0.001
**Lactate/albumin ratio (as a continuous variable)**			
	**Odds ratio**	**95% confidence interval**	***P***
**In-hospital mortality**			
Model 1	2.89	2.23-3.75	<0.001
Model 2	2.95	2.25-3.86	<0.001
Model 3	2.58	1.88-3.54	<0.001
Model 4	2.56	1.86-3.53	<0.001
**30-day mortality**			
Model 1	2.95	2.27-3.84	<0.001
Model 2	2.99	2.28-3.92	<0.001
Model 3	2.61	1.90-3.59	<0.001
Model 4	2.59	1.87-3.58	<0.001

Model 1 = Non-adjusted model; Model 2 = age + gender; Model 3 = model 2 + (laboratory data); Model 4 = model 3 + (comorbidities).

### Survival analysis of pediatric AKI patients with different LAR levels

**Fig 3:**
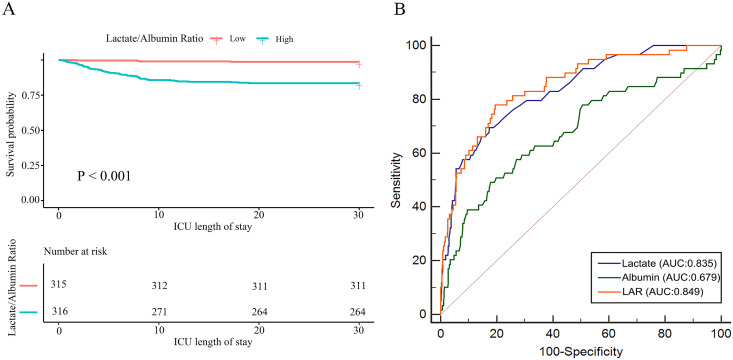
Kaplan-Meier analysis and receiver operating characteristic (ROC) analysis. **(A)** Survival analysis of pediatric ackte kidney injury (AKI) patients with different lactate/albumin ratio (LAR) levels; **(B)** Predictive value of LAR for hospital mortality in pediatric AKI patients.

The Kaplan-Meier curve illustrating the relationship between different LAR levels and 30-day mortality in pediatric AKI patients is shown in Fig 3A. Visual inspection of the curves suggests the survival difference between the groups was most pronounced within the initial phase (approximately the first 20 days). The log-rank test comparing the survival distributions over the entire 30-day period demonstrated a significantly lower overall survival probability for patients in the high LAR group compared to those in the low LAR group (*P* < 0.001).

### Predictive value of LAR for hospital mortality in pediatric AKI patients

As shown in Fig 3B, LAR demonstrated good predictive ability for hospital mortality, with an area under the curve (AUC) of 0.849 (95% CI 0.819–0.876), which was higher than that of lactic acid (AUC 0.835) and albumin (AUC 0.679). At the optimal threshold of 0.80, the sensitivity was 80.42%, specificity was 77.97%, accuracy was 80.19%, positive predictive value was 97.25%, negative predictive value was 29.11%, positive likelihood ratio was 3.982, negative likelihood ratio was 0.274, and diagnostic odds ratio was 14.533. These results indicate that LAR has high efficacy in distinguishing between patients who died during hospitalization and those who survived. The diagnostic detection requirement was 1.71, further demonstrating the high clinical efficiency of this model.

### Sensitivity analysis

Beyond these primary analyses, we further assessed the internal robustness and generalizability of LAR’s predictive performance. A Bootstrapping resampling procedure with 500 iterations yielded an average AUC for hospital mortality of 0.849 (95% CI 0.799–0.900), which aligned closely with the primary analysis. The mean optimal threshold derived from Bootstrapping was 0.80 (95% CI 0.64–1.17), with average sensitivity, specificity, and accuracy of 78.0% (95% CI 65.7%–89.2%), 79.9% (95% CI 69.9%–90.0%), and 80.3% (95% CI 71.5%–90.9%), respectively. These Bootstrapping results demonstrate the statistical stability of our prognostic efficacy estimates for LAR. Additionally, a random 30% subsample (n = 190) from the total dataset, when evaluated using the optimal LAR cutoff of 0.80 determined from the full dataset, showed an AUC of 0.881 (95% CI 0.806–0.943) for predicting hospital mortality. In this subsample, the sensitivity was 84.2% (95% CI 73.0%–100.0%), specificity was 80.1% (95% CI 62.4%–91.6%), and accuracy was 78.8% (95% CI 65.8%–91.6%). These findings from the random subsample further support the consistency of LAR’s predictive ability and the identified threshold across different data subsets within our cohort.

To further assess the reliability of our primary findings concerning the association between LAR and mortality, we conducted additional sensitivity analyses by systematically excluding two specific patient subgroups. These analyses involved repeating the multivariate logistic regression models and the ROC curve analysis for hospital mortality after systematically excluding two subgroups: (1) patients who received albumin replacement therapy within the first 24 hours of PICU admission (n = 22), and (2) patients with a diagnosis of liver dysfunction (n = 113). The results from both sensitivity analyses were consistent with those of the primary analysis (S2 and S3 Table). After excluding patients receiving early albumin substitution, LAR (as a continuous variable) remained a significant independent predictor of hospital mortality (Adjusted OR 2.71, 95% CI 1.93–3.80) and 30-day mortality (Adjusted OR 2.72, 95% CI 1.92–3.84). Similarly, when patients with liver dysfunction were excluded, the significant association between LAR and hospital mortality (Adjusted OR 2.86, 95% CI 1.84–4.43) and 30-day mortality (Adjusted OR 2.94, 95% CI 1.86–4.64) persisted. Furthermore, the predictive performance of LAR for hospital mortality remained good in both sensitivity analyses, with AUC values similar to the primary analysis (AUC 0.845, 95% CI 0.814–0.873 after excluding albumin substitution; AUC 0.810, 95% CI 0.774–0.843 after excluding liver dysfunction).

## Discussion

This study conducted a retrospective analysis of 631 pediatric patients with AKI, marking the first comprehensive evaluation of the LAR in predicting the prognosis of pediatric AKI. The results demonstrated a significant positive correlation between LAR and hospital mortality in pediatric AKI patients. Multivariate logistic regression analysis identified LAR as an independent predictor of hospital mortality, with this finding remaining consistent after adjusting for various potential confounders. ROC curve analysis further validated LAR’s strong predictive capacity for prognosis, suggesting that it could be an effective tool for risk assessment in pediatric AKI.

The association between elevated LAR and poor prognosis may stem from several pathophysiological mechanisms. First, increased lactate levels typically reflect insufficient tissue perfusion and an imbalance between oxygen supply and demand, which are closely linked to microcirculatory dysfunction and metabolic disturbances at the cellular level [ 16,17]. Second, hypoalbuminemia not only indicates poor nutritional status but may also lower colloid osmotic pressure, leading to tissue edema and impairing vital organ functions [18]. Furthermore, albumin performs crucial functions such as antioxidant protection, molecular transport, and toxin clearance. Lower albumin levels may exacerbate inflammation and oxidative stress [19, 20]. By combining lactate and albumin ratios, we assess both tissue perfusion and the body’s compensatory mechanisms, potentially explaining why LAR provides superior predictive value compared to lactate or albumin alone.

The results of this study align with previous research on prognosis prediction in adult AKI. For instance, Zhu et al. conducted a retrospective analysis of 4666 adult AKI patients and found a significant correlation between LAR and hospital mortality when treated as a continuous variable (HR 1.22, 95% CI 1.18–1.26). ROC analysis indicated that LAR effectively predicted hospital mortality (AUC 0.717) and ICU mortality (AUC 0.733) [21]. Similarly, Gharipour et al. evaluated the prognostic value of LAR for ICU mortality in adults, where the ROC-AUC for predicting ICU mortality was 0.69 (95% CI 0.67–0.70), with a cutoff value of 1.01 [22]. However, our study found that the optimal LAR cutoff for pediatric patients was significantly lower than for adults (0.80 vs 1.01), and its prognostic predictive ability was stronger (AUC 0.85 vs 0.69). This difference may be attributed to several factors. Children and adults differ in metabolic characteristics, with children having higher basal metabolic rates and more robust organ compensation mechanisms [23]. Additionally, the causes of AKI in children often differ from those in adults, with infections and prerenal factors being more prevalent. Furthermore, children generally receive earlier interventions and more intensive monitoring, which may enhance the predictive performance of prognostic markers [24].

Widely used scoring systems in pediatric intensive care, such as the Pediatric Risk of Mortality IV, Pediatric Index of Mortality 3, and Pediatric Logistic Organ Dysfunction 2 scores, have proven effective in assessing the severity and prognosis of septic children [25–28]. While valuable, these comprehensive scoring systems necessitate collecting and integrating data from multiple indicators. This inherent complexity, along with potential challenges related to data availability for all components in fast-paced clinical environments, can sometimes pose practical difficulties for routine or rapid bedside risk assessment. In contrast, LAR, relying on just two commonly measured laboratory tests, presents a simpler and potentially more readily applicable approach for prognostic risk stratification in this context. With an optimal cutoff of 0.80, LAR demonstrates good diagnostic performance, including a sensitivity of 80.42%, specificity of 77.97%, and accuracy of 80.19%. In emergency and intensive care settings, LAR could serve as an important tool for risk stratification [29].

Despite its strengths, this study has several limitations. First, as a single-center retrospective study, it may be subject to selection bias, potentially affecting the generalizability of the findings. Second, due to limitations in the available database, certain confounders, such as body weight, detailed nutritional status scores, diagnostic information regarding sepsis, and medication data, could not be accounted for. Additionally, this study did not evaluate the impact of LAR-guided treatment or LAR’s role in renal recovery, which warrant investigation in future prospective studies. Nevertheless, the key findings from this study provide important clinical insights and practical value.

## Conclusion

In conclusion, this study, to our knowledge, is among the first to explore the significant value of LAR in predicting the prognosis of pediatric AKI patients. The results suggest that LAR is significantly associated with hospital mortality, appears to act as an independent prognostic factor, and demonstrates promising predictive accuracy. As a simple, cost-effective, and readily accessible indicator, LAR holds potential for clinical application. This index could potentially aid in the early identification of high-risk children, which may allow for the optimization of treatment strategies and improvement in outcomes. Future multicenter prospective studies are needed to further validate the prognostic value of LAR, particularly regarding its effectiveness in predicting the prognosis of pediatric AKI patients across different regions and healthcare settings.

## Supporting information

S1 Table Diagnostic criteria for complications. (DOCX)

S2 Table Stepwise expansion of the multivariate logistic regression model with sensitivity analysis excluding patients receiving albumin within the initial 24 hours of PICU admission. (DOCX)

S3 Table Stepwise expansion of the multivariate logistic regression model with sensitivity analysis excluding liver dysfunction. (DOCX)
