# Supplementary material for: The relationship between lactate/albumin ratio and prognosis in children with acute kidney injury
Source: PLoS One. 2025 Aug 1;20(8):e0329453. doi: 10.1371/journal.pone.0329453 (PMC12316205; doi:10.1371/journal.pone.0329453)
Supplement: S1 Table — (DOCX) [file pone.0329453.s001.docx]

**S1 Table. Diagnostic criteria for complications.**

| **Complications** | **Diagnostic criteria** |
| --- | --- |
| Hypertension | Systolic pressure > 120 mmHg; diastolic pressure > 80 mmHg. |
| Anemia | 1-4 months, < 90 g/L; 4-6 months, < 100 g/L; 6-60minths, < 110 g/L; 60-144months, < 115 g/L; >144 months, < 120 g/L. |
| Pneumonia | Including pneumonia caused by various pathogens. |
| Diabetic ketoacidosis | Blood glucose > 11 mmol/L; venous blood pH < 7.3 or serum bicarbonate < 15 mmol / L; presence of ketone bodies in blood or urine. |
| Systemic inflammatory response syndrome | Meet two or more of the following conditions:  Body temperature: > 38°C or < 36°C.  Heart rate: > 90 beats per minute.  Respiration: > 20 breaths per minute or hyperventilation, with PaCO₂ < 32 mmHg.  Blood routine: White blood cell count > 12×10⁹/L or < 4×10⁹/L, or immature granulocytes > 10%. |
| Liver dysfunction | Total bilirubin > 68.4mmol/L or alanine aminotransferase elevation more than two times the upper value. |
