# Supplementary material for: The relationship between lactate/albumin ratio and prognosis in children with acute kidney injury
Source: PLoS One. 2025 Aug 1;20(8):e0329453. doi: 10.1371/journal.pone.0329453 (PMC12316205; doi:10.1371/journal.pone.0329453)
Supplement: S2 Table — (DOCX) [file pone.0329453.s002.docx]

**S2 Table. Stepwise expansion of the multivariate logistic regression model with sensitivity analysis excluding patients receiving albumin within the initial 24 hours of PICU admission.**

|  | **Lactate/albumin ratio (>0.50)** | | |
| --- | --- | --- | --- |
|  | **Odds ratio** | **95% confidence interval** | ***P*** |
| **In-hospital mortality** |  |  |  |
| Model 1 | 9.52 | 4.00-22.63 | <0.001 |
| Model 2 | 9.55 | 4.01-22.70 | <0.001 |
| Model 3 | 8.34 | 3.38-20.57 | <0.001 |
| Model 4 | 7.17 | 2.85-18.08 | <0.001 |
| **30-day mortality** |  |  |  |
| Model 1 | 14.01 | 4.98-39.45 | <0.001 |
| Model 2 | 14.01 | 4.97-39.46 | <0.001 |
| Model 3 | 11.84 | 4.08-34.39 | <0.001 |
| Model 4 | 10.11 | 3.41-29.97 | <0.001 |
|  | | | |
|  | **Lactate/albumin ratio (as a** **continuous variable)** | | |
|  | **Odds ratio** | **95% confidence interval** | ***P*** |
| **In-hospital mortality** |  |  |  |
| Model 1 | 2.93 | 2.24-3.84 | <0.001 |
| Model 2 | 2.95 | 2.25-3.86 | <0.001 |
| Model 3 | 2.64 | 1.91-3.63 | <0.001 |
| Model 4 | 2.71 | 1.93-3.80 | <0.001 |
| **30-day mortality** |  |  |  |
| Model 1 | 3.00 | 2.29-3.95 | <0.001 |
| Model 2 | 3.02 | 2.30-3.97 | <0.001 |
| Model 3 | 2.67 | 1.93-3.70 | <0.001 |
| Model 4 | 2.72 | 1.92-3.84 | <0.001 |

Model 1 = Non-adjusted model;

Model 2 = age + gender;

Model 3 = model 2 + (laboratory data);

Model 4 = model 3 + (comorbidities).
