# Supplementary material for: The relationship between lactate/albumin ratio and prognosis in children with acute kidney injury
Source: PLoS One. 2025 Aug 1;20(8):e0329453. doi: 10.1371/journal.pone.0329453 (PMC12316205; doi:10.1371/journal.pone.0329453)
Supplement: S3 Table — (DOCX) [file pone.0329453.s003.docx]

**S3 Table. Stepwise expansion of the multivariate logistic regression model with sensitivity analysis excluding liver dysfunction.**

|  | **Lactate/albumin ratio (>0.50)** | | |
| --- | --- | --- | --- |
|  | **Odds ratio** | **95% confidence interval** | ***P*** |
| **In-hospital mortality** |  |  |  |
| Model 1 | 5.94 | 2.40-14.69 | <0.001 |
| Model 2 | 6.01 | 2.42-14.92 | <0.001 |
| Model 3 | 5.96 | 2.31-15.40 | <0.001 |
| Model 4 | 6.09 | 2.34-15.86 | <0.001 |
| **30-day mortality** |  |  |  |
| Model 1 | 8.97 | 3.08-26.11 | <0.001 |
| Model 2 | 9.07 | 3.11-26.45 | <0.001 |
| Model 3 | 8.43 | 2.80-25.41 | <0.001 |
| Model 4 | 8.70 | 2.86-26.53 | <0.001 |
|  | | | |
|  | **Lactate/albumin ratio (as a continuous variable)** | | |
|  | **Odds ratio** | **95% confidence interval** | ***P*** |
| **In-hospital mortality** |  |  |  |
| Model 1 | 2.61 | 1.85-3.67 | <0.001 |
| Model 2 | 2.77 | 1.92-3.99 | <0.001 |
| Model 3 | 2.70 | 1.76-4.14 | <0.001 |
| Model 4 | 2.86 | 1.84-4.43 | <0.001 |
| **30-day mortality** |  |  |  |
| Model 1 | 2.73 | 1.92-3.88 | <0.001 |
| Model 2 | 2.95 | 2.02-4.32 | <0.001 |
| Model 3 | 2.78 | 1.78-4.34 | <0.001 |
| Model 4 | 2.94 | 1.86-4.64 | <0.001 |

Model 1 = Non-adjusted model;

Model 2 = age + gender;

Model 3 = model 2 + (laboratory data);

Model 4 = model 3 + (comorbidities).
